# Composite of bentonite and cyclodextrin as an efficient catalyst for promoting chemical transformations in aqueous media

**DOI:** 10.1038/s41598-021-84349-9

**Published:** 2021-03-03

**Authors:** Fatemeh Koohestani, Samahe Sadjadi, Majid Heravi

**Affiliations:** 1 Gas Conversion Department, Faculty of Petrochemicals, Iran Polymer and Petrochemical Institute, PO Box 14975-112, Tehran, Iran; 2 Department of Chemistry, School of Physic and Chemistry, Alzahra University, PO Box 1993891176, Vanak, Tehran Iran

**Keywords:** Catalysis, Green chemistry

## Abstract

Combining the encapsulating capability of cyclodextrin and instinctive features of bentonite clay, a versatile metal free catalyst has been developed that could promote various chemical reactions such as Knoevenagel condensation, synthesis of xanthan and octahydroquinazolinones in aqueous media under ultrasonic irradiation. To prepare the catalyst, bentonite was Cl-functionalized and then reacted with isatin and guanidine successively to furnish amino functionalized bentonite. The latter then reacted with tosylated cyclodextrin. The resultant catalytic composite was characterized via XRD, SEM, EDS, BET, elemental mapping analysis, TGA and FTIR. The catalytic activity tests approved excellent activity of the catalyst as well as broad substrate scope. Notably, the catalyst could be simply recovered and reused for several reaction runs. Moreover, the activity of the composite was superior to that of its components.

## Introduction

Clays are one of the mostly attractive natural materials that have been extensively applied for the catalysis. Apart from their low cost and availability, the diversity in their structure and chemical composition as well as their high stability make them valuable choices both as catalysts and supporting materials. Among layered clays, bentonite, Bent, is one of the mostly utilized one for developing heterogeneous catalysts. This clay possesses empirical formula of Rx(H_2_O)_4_{(Al_2_-x,Mgx)_2_[(Si,Al)_4_O_10_](OH)_2_}, where R refer to the exchangeable cations of alkali and alkali-earth metals between the layers. Moreover, there are numerous –OH functionalities on Bent that allow introduction of other functional moieties on Bent^1–4^.

Conjugation of carbohydrates and clays is a well-established strategy to produce composites that benefits from the properties of both components^5,6^. Among various carbohydrate, cyclodextrins, CDs, have attracted immense attention due to their cone shapes that allow CDs to act as nanoreactors for hosting guests with appropriate chemistry and sizes^7–19^. Using this feature of CD, many of chemical processes with hydrophobic reagents were accomplished in aqueous media. In fact, CDs were applied as shuttles for accommodating the hydrophobic reagents in their hydrophobic cavities and carrying them into the hydrophilic media.

Using ultrasonic irradiation (US) for accomplishing chemical transformations is a well-known protocol that can accelerate the reaction rate and provide environmentally benign protocols^20^. Ultrasonic irradiations cause cavitation phenomenon. In more detail, US resulted in the formation of bubbles with high pressure and temperature. The collapse of these bubbles generates active spots for promoting the reactions^21^.

**Figure 1:**
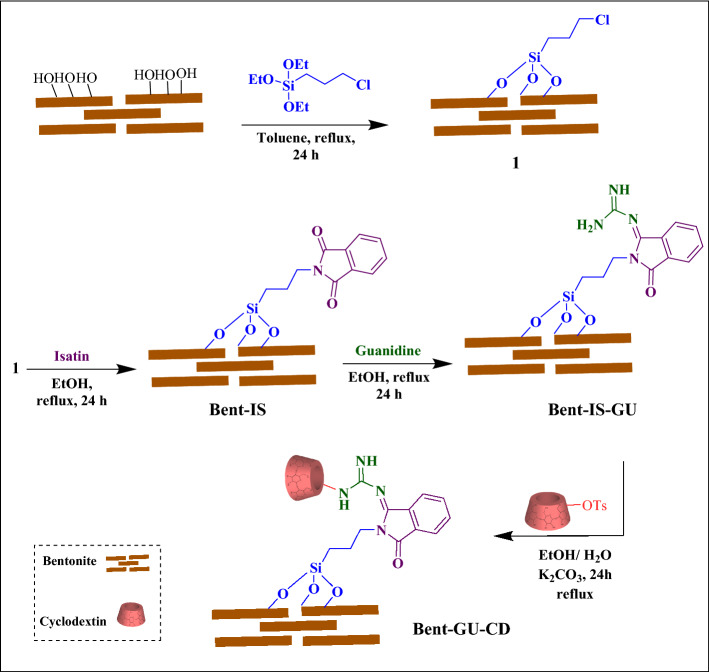
Schematic diagram for the synthesis of Bent-Gu-CD.

**Figure 2:**
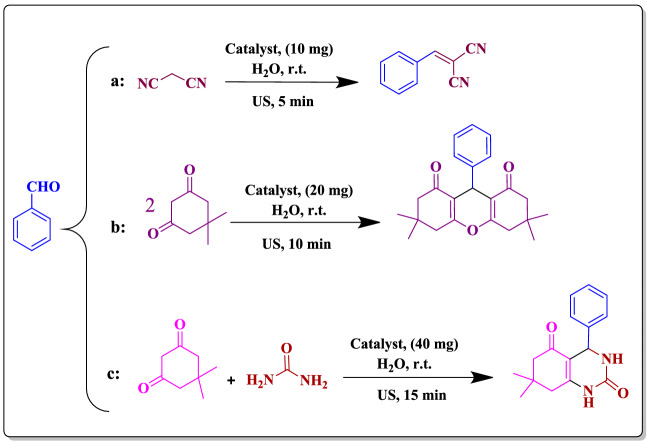
Model reaction of (**a**) Knoevenagel condensation, (**b**) synthesis of xanthan and (**c**) octahydroquinazolinones.

In pursuit of our research on employing natural compounds for the fabrication of effective and low-cost catalytic systems^22–24^, herein we wish to present a versatile metal-free catalyst for performing chemical transformations in aqueous media under ultrasonic irradiation. For the fabrication of the catalyst, Bent was Cl-functionalized and then reacted successively with isatin and guanidine. The resultant compound was then tolerated reaction with the as-prepared tosylated CD to furnish a composite that possesses both clay and carbohydrate in its framework (Fig. 1). One reason for using these two components is that they are natural, biocompatible and non-toxic materials that are relatively low-cost. The second reason is related to the properties of these two components. According to the literature, cyclodextrin that is a cyclic carbohydrate with a hydrophobic cavity and hydrophilic surface can encapsulate the hydrophobic guests and then transfer them in aqueous media. In other word, it can act as a molecular shuttle^7–20^. The reason for using bentonite is that this clay has intrinsic catalytic activity. Moreover, this clay is locally abundant and very low-cost. On the other hand, similar to other clays it benefits from high chemical and thermal stability. The performance of the catalyst for three chemical transformations, Knoevenagel condensation as well as synthesis of xanthan and octahydroquinazolinones was examined (Fig. 2). Moreover, the substrate scope and recyclability of the catalyst for the latter reaction were evaluated.

## Result and discussion

### Validation of the structure of Bent-Gu-CD

**Figure 3:**
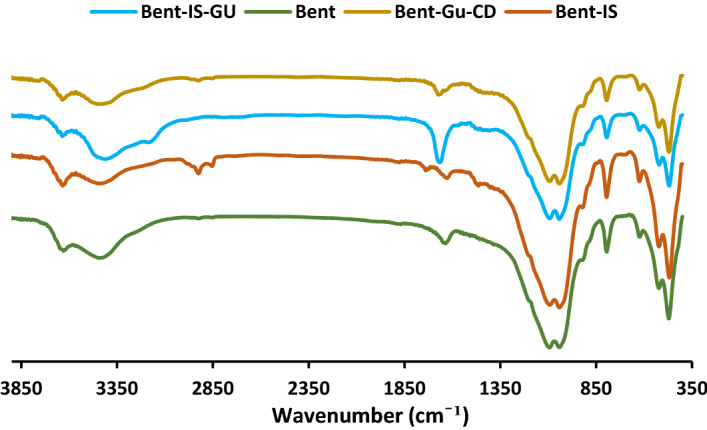
FTIR spectra of Bent, Bent-IS, Bent-IS-Gu and Bent-Gu-CD.

Bent, Bent-IS, Bent-IS-Gu and Bent-Gu-CD (Fig. 1) were all characterized by FTIR spectroscopy (Fig. 3). According to the literature, the absorbance bands of Bent are the bands at 1032 cm^−1^ (Si–O–Si), 795 cm^−1^ (Si–O), 1636 cm^−1^ (H_2_O), 3631–3425 cm^−1^ (–OH), and 526 cm^−1^ (Al–O–Si)^1,25,26^. These characteristic bands are observable in the spectra of Bent-IS, Bent-IS-Gu and Bent-Gu-CD, approving the fact that Bent structure is maintained in each preparation step. In the FTIR spectrum of Bent-IS, an additional band appeared at 1742 cm^−1^ that is representative of –C=O in the IS structure. In the FTIR spectrum of Bent-IS-Gu, the band at 1685 cm^−1^ can be ascribed to the –C = N functionality in GU. FTIR spectrum of the catalyst is similar to that of Bent-IS-Gu. This issue is due to the fact that the characteristic bands of CD overlapped with those of Bent-IS-Gu.

**Figure 4:**
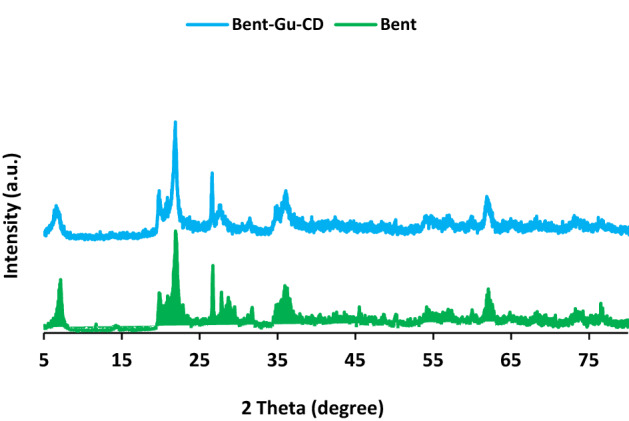
XRD patterns of Bent and Bent-Gu-CD.

XRD is a powerful analysis that can establish whether Bent instinct structure will be maintained in the course of functionalization. Figure 4 illustrated the comparison of XRD patterns of Bent and Bent-Gu-CD. It can be corroborated that the two patterns are similar and exhibit the characteristic peaks of Bent at 2θ = 7°, 20.8°, 21.9°, 26.6°, 27.7°, 31.7°, 36°, 50°, 62°, 73.5° and 76°^27,28^. This result clearly confirmed that Bent was stable and did not structurally alter during surface modification.

**Figure 5:**
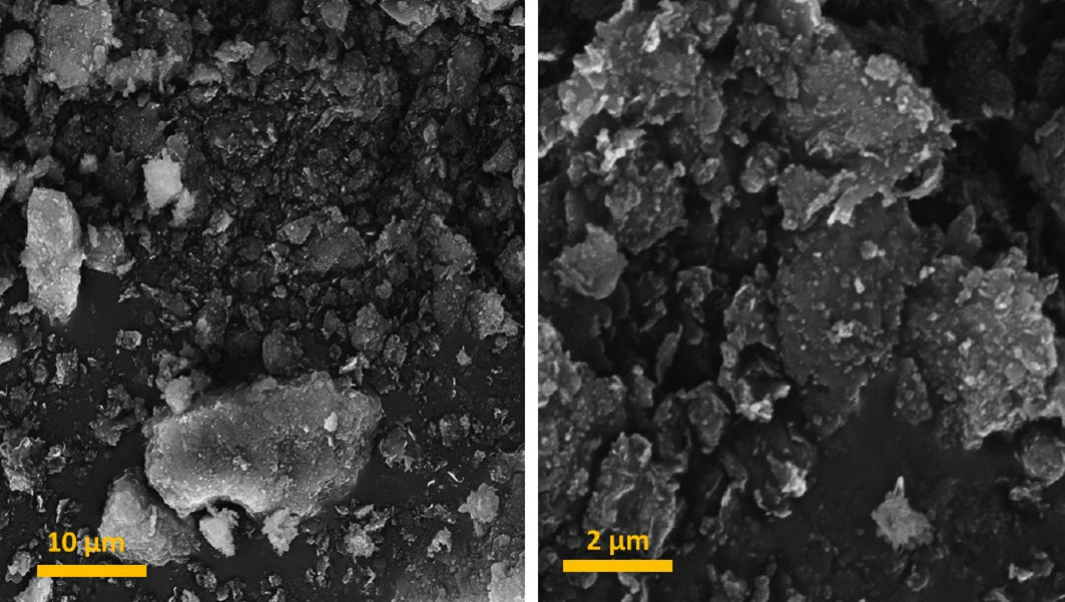
SEM images of the Bent-Gu-CD.

SEM images of Bent-Gu-CD is provided in Fig. 5. As shown, the catalyst possesses an aggregated morphology that is distinguished from the morphology of the used Bent (Supplementary Fig. S1).

**Figure 6:**
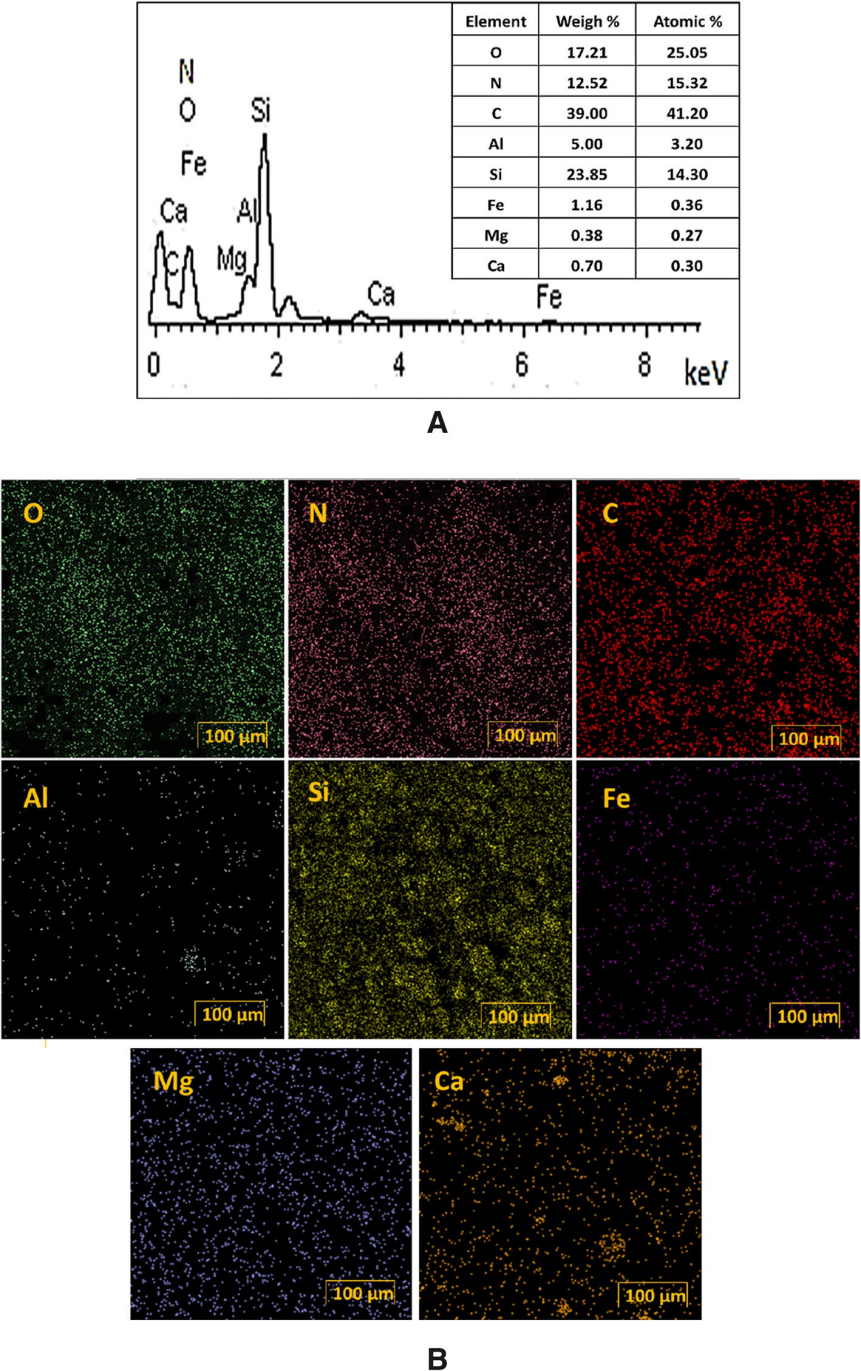
(**A**) EDS and (**B**) Elemental mapping analysis of Bent-Gu-CD.

The results of EDS analysis of Bent-Gu-CD is presented in Fig. 6A. According to the literature, Si, Fe, Mg, O, Al and Ca are indicative of Bent structure^29^. C and O atoms can be attributed to the CD structure. Moreover, N, C and O atoms can be ascribed to IS-Gu. High dispersion of C and N atoms in the elemental mapping analysis, Fig. 6B, approved homogeneous functionalization of Bent with the organic functionality.

TG analysis of the catalyst, Supplementary Fig. S2, indicated that apart from the known weight losses of Bent, i.e. weight losses as a result of dehydration (at ~ 200 °C) and Bent degradation (~ 460–560 °C), an additional weight loss at 280 °C can be discerned that is due to the degradation of the incorporated organic modifying agents. Using TGA, the content of CD in Bent-Gu-CD was estimated to be 7 wt%.

Using BET, the specific surface area of Bent-Gu-CD was estimated as ~ 6 m^2^ g^−1^. This value was lower than that of Bent (40.62 m^2^).

### Catalyst activity

Using Bent that is a natural clay with instinct catalytic activity and CD that can act as a molecular shuttle, a novel metal free catalyst, Bent-Gu-CD, is fabricated. It was assumed that this catalyst can serve as a versatile catalyst with utility for promoting various chemical transformations in aqueous media. To examine this postulation, a model Knoevenagel condensation was first carried out through reaction of benzaldehyde and malononitrile in the presence of scant amount (10 mg) of Bent-Gu-CD in H_2_O, Fig. 2. Furthermore, to accelerate the reaction and provide environmentally benign procedure, the reaction was performed under ultrasonic irradiation. Gratifyingly, it was found that under Bent-Gu-CD catalysis, the reaction proceeded rapidly and resulted in 100% conversion and yield after 5 min.

Encouraged by high activity of the catalyst, more complicated reaction, synthesis of a model xanthan, was examined via ultrasonic-assisted reaction of benzaldehyde, dimedone and catalytic dosage of Bent-Gu-CD (20 mg) in aqueous media at ambient temperature. Interestingly, it was shown that 100% conversion and yield were achieved after 10 min.

To further establish the diversity of the catalyst, ultrasonic-assisted synthesis of octahydroquinazolinones in aqueous media was appraised. To this purpose, the catalyst dosage was optimized (Supplementary Table S1). The results confirmed that using 40 mg of Bent-Gu-CD, the model reaction, reaction of benzaldehyde, dimedone and urea, proceeded to give 100% conversion and yield after 15 min.

Next, the substrate scope was examined for the synthesis of octahydroquinazolinones (Table 1). As tabulated, the presented protocol could be generalized to various aldehydes, including aldehydes with different groups and heterocyclic aldehydes. In all cases, high reaction yield was achieved in a very short reaction time. However, it can be recognized that use of heterocyclic aldehydes resulted in slightly lower yield. On the other hand, comparison of the aldehydes with same functional groups indicated that the position of the functionality can affect the obtained yield, Table 1, entries 1 and 2. As shown, presence of the functional group on *para* position was favorable and led to superior yield. This issue can be rationalized by considering the fact that encapsulation of aldehydes substituted on *para* position is more facile that other positions.

**Table 1:** Synthesis of octahydroquinazolinone derivatives catalyzed by Bent-Gu-CD under ultrasonic irradiation condition.


No.	Substrate	Product	Yield^a^ (%)	M.P °C	References
1			100	289–291	290–291^30^
2			98	297–300	300–301^31^
3			100	300–303	302–304^32^
4			90	281–282	281–283^33^
5			95	298–280	297–299^34^
6			90	> 300	> 300^30^

Reaction condition: Substrate (1 mmol), urea (1 mmol), dimedone (1 mmol), H_2_O (15 mL) at 25 °C in 15 min under ultrasonic irradiation.

^a^Isolated yield.

### Elucidating the merit of conjugation of Bent and CD

Next, the merit of conjugation of Bent and CD was appraised by comparing the efficiency of Bent, CD, and Bent-IS-GU with that of Bent-Gu-CD for the synthesis of the model octahydroquinazolinone. Performing the reaction under optimum condition in the presence of Bent indicated that only 40% yield of the product was furnished after 15 min. Regarding the efficiency of CD, the obtained yield was 35%. Apart from low yield, the homogeneous nature of CD was troublesome. In the case of Bent-IS-Gu, the catalytic efficiency improved to 70%. This can be due to the role of amino functionalities on activation of the reagents. As mentioned before, the activity of Bent-Gu-CD was superior to all of the control samples and reached to 100%, implying that conjugation of amino-functionalized Bent and CD is favorable for the reaction.

### Proposed mechanism

**Figure 7:**
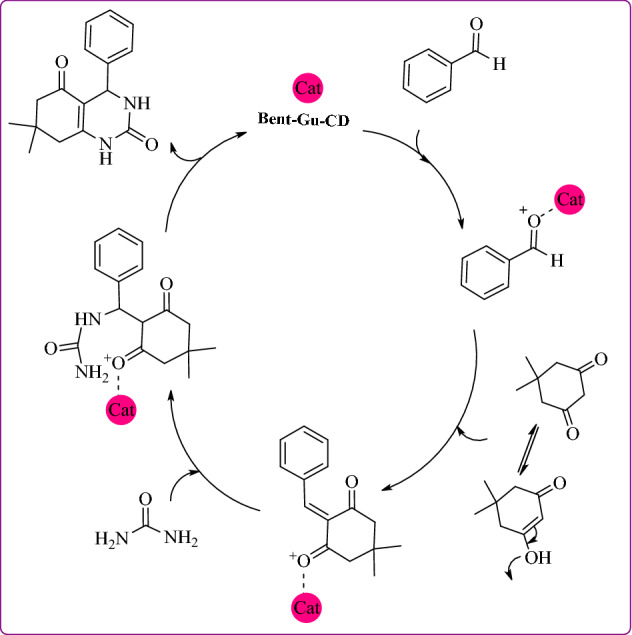
Proposed mechanism for the synthesis of octahydroquinazolinones.

Regarding the reaction mechanism of formation of octahydroquinazolinones, it can be proposed that aldehyde was first encapsulated in CD cavity and transferred into the aqueous media. Then, it was activated by the catalyst and underwent reaction with enol form of dimedone. Subsequently, the formed intermediate tolerated dehydration and then reacted with urea. The final product was furnished through cyclization and dehydration (Fig. 7)^31^.

### Study of nature of catalysis: Sheldon test

To validate the nature of catalysis and establish the real heterogeneity of Bent-Gu-CD, Sheldon test has been carried out^35^ to perform this test, the reaction of synthesis of the model octahydroquinazolinone has been halted after 5 min and the catalyst has been separated. Then, the reaction was continued in the absence of the catalyst. Measurement of the yield of the reaction before and after catalyst removal showed that after separation of the catalyst the reaction did not proceed. This observation approved heterogeneous nature of the catalysis.

### Evaluation of recyclability of Bent-Gu-CD

**Figure 8:**
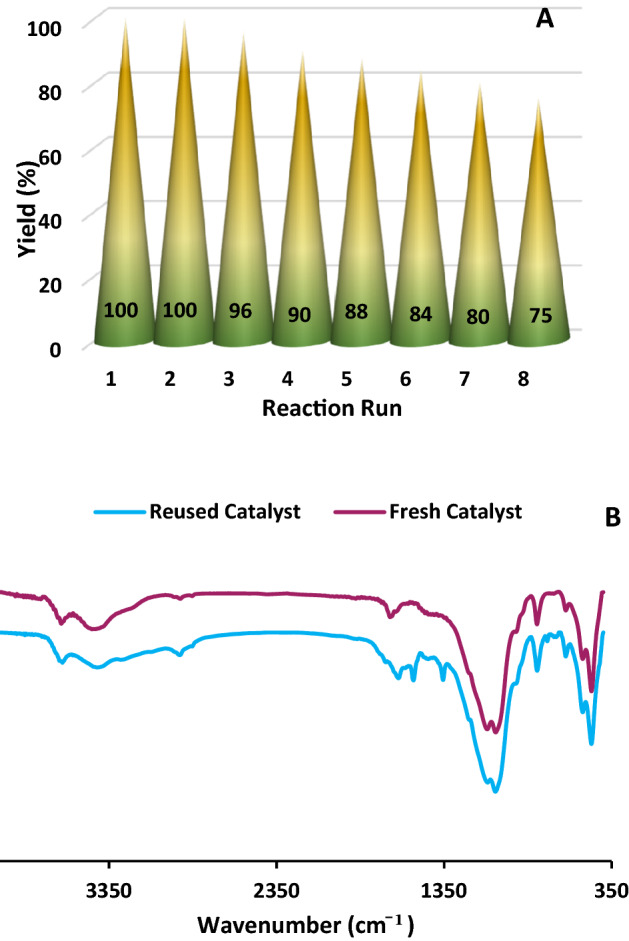
(**A**) The recyclability of the Bent-Gu-CD and (**B**) FTIR spectrum of the recycled catalyst after eight runs in synthesis of octahydroquinazolinones under optimum reaction condition.

To evaluate the recyclability of the developed Bent-Gu-CD, the performance of the recycled catalyst for the synthesis of octahydroquinazolinones was examined. In this line, the recovered Bent-Gu-CD from the first reaction run was thoroughly washed and dried and reused for the second run of the same process under exact similar condition. As shown in Fig. 8A, Bent-Gu-CD maintained its activity for the second run. Recovering-reuse cycle was repeated up to eight runs. As illustrated in Fig. 8A, after the second run, the yield of the reaction slightly decreased and reached to 75% at eighth run.

To find out the origin of the observed loss of the activity of Bent-Gu-CD upon recycling, FTIR spectroscopy was employed. As shown in Fig. 8B, the comparison of the FTIR spectra of recycled and fresh Bent-Gu-CD indicated that although the structure of Bent-Gu-CD was preserved, some additional absorbance bands as well as band broadening were discerned. This observation can be ascribed to the deposition of organic substrates on the surface of Bent-Gu-CD. In fact, the coverage of the active spots on Bent-Gu-CD can justify the observed reduction of the catalytic activity.

## Experimental section

### Materials and instrument

For the fabrication of the catalyst the following chemicals were applied: (3-chloropropyl) trimethoxysilane (CPTES), isatin (IS), triethylamine (TEA), guanidine (Gu), β-CD, *p*‐toluenesulfonyl chloride (*p*‐TsCl), K_2_CO_3_, toluene, EtOH and Bent. Bent was obtained from Madan Kavan Co. Iran. The other reagents were purchased from Sigma-Aldrich. For performing the ultrasonic-assisted chemical transformations, benzaldehyde, malononitrile, dimedone and urea (provided from Sigma-Aldrich) have been used.

Bent-Gu-CD synthesis was verified by Fourier transform infrared (FTIR), Thermo gravimetric analysis (TGA), X-ray diffraction (XRD), scanning electron microscope (SEM), energy dispersive spectroscopy (EDS) and elemental mapping analysis. The FTIR spectrum were collected using PERKIN-ELMER Spectrum 65. For TGA, METTLER TOLEDO apparatus was employed. The tests were carried out under N_2_ atmosphere at heating rate of 10 °C min^−1^. XRD pattern of Bent-Gu-CD was gathered via Siemens, D5000 apparatus with Cu Kα as a radiation source. SEM /EDX analyses were carried out on MIRA 3 TESCAN-XMU. Brunauer Emmett Teller (BET) analysis was carried out via a Belsorp Mini II apparatus. Degassing was performed at 150 °C for 3 h.

### Catalysts preparation

#### Synthesis of Bent-Cl

Initially, the surface of Bent was functionalized with CPTES. To this purpose, Bent (4 g) was dispersed in dry toluene (70 mL) under constant stirring, and then 3 mL of CPTES was injected drop by drop into the Bent suspension. Subsequently, the obtained mixture was continuously stirred under reflux condition at 110 °C overnight. In the next step, the product, Bent-Cl, was filtered, washed with toluene (30 mL) repeatedly and dried overnight at 70 °C.

#### Synthesis of Bent-IS

A solution of IS (15 mmol in 20 mL of EtOH) was added to the homogeneous suspension of Bent-Cl (3 g, in EtOH). Then, TEA (20 mL) was added slowly into the above mention suspension, and the resulting mixture was refluxed for 24 h. Afterward, the reaction mixture was subjected to centrifugation and the resulting solid, Bent-IS, was washed with MeOH (2 × 10 mL) to remove the unreacted materials and dried in an oven at 70 °C overnight.

#### Synthesis of Bent-IS-Gu

Bent-IS (3 g) was dispersed in EtOH (80 mL) and magnetically stirred for 30 min to achieve a homogenous suspension. Next, to the Bent-IS suspension, Gu (2 g) was added, and the subsequent mixture was refluxed for 24 h at 100 °C. Finally, the obtained product, Bent-Gu, was filtered out by centrifugation and then washed with EtOH. The obtained Bent-Gu was dried at 60 °C overnight.

#### Synthesis of Bent-Gu-CD

Bent-Gu-CD was prepared in two steps. In the first step, CD was tosylated with *p*-TsCl. To this purpose, the mixture of CD (15.86 mmol) and *p*-TsCl (7.9 mmol) in pyridine (200 mL) was kept in the refrigerator at 0 °C for 48 h. Afterward, an oily product was furnished by adding distilled water (75 mL). Subsequently, cold water was poured to the oily product and the solid CD-OTs, was purified through recrystallization from water.

In second step, Bent-Gu was modified with CD. In this regard, Bent-Gu (2 g) suspension in H_2_O/ EtOH (2:1, 60 mL) was dispersed via ultrasonic irradiation (120 W, 30 min). Then, the solution of CD-OTs (1.5 g) and K_2_CO_3_ (0.1 g) in H_2_O (15 mL) was injected to the so-called suspension and the resultant mixture was refluxed at 100 °C for 24 h. At the end, the reaction mixture was subjected to centrifugation and, the solid Bent-Gu-CD was washed with H_2_O (10 × 20 mL) and EtOH (3 × 10 mL) and dried in an oven overnight (Fig. 1).

### Examining the catalytic activity: ultrasonic-assisted chemical transformations

#### Knoevenagel condensation

In a typical procedure, benzaldehyde (1 mmol), malononitrile (1.2 mmol) and Bent-Gu-CD (10 mg) were mixed in H_2_O (10 mL) in a reaction vessel. The reaction proceeded under ultrasonic irradiation (20 kHz frequency for 5 min) at room temperature. After the completion of the reaction (traced by thin layer chromatography), Bent-Gu-CD was separated via centrifuge and then washed several times with MeOH (Supplementary Figs. S3 and S4).

#### Synthesis of Xanthan

Typically, a mixture of benzaldehyde (1 mmol), dimedone (2 mmol), H_2_O (15 mL) and Bent-Gu-CD (20 mg) were sonicated at 25 °C for 10 min. The ultrasonic apparatus was equipped with thermal sensor and in the case of temperature evolution, the temperature was controlled with cold water. The reaction was followed by TLC. Then, the reaction mixture diluted with MeOH and the catalyst was filtered off. The recovered Bent-Gu-CD was washed and dried at 100 °C in an oven (Supplementary Figs. S5 and S6).

#### Synthesis of octahydroquinazolinones

In a typical process, a mixture of benzaldehyde (1 mmol), dimedone (1 mmol), urea (1 mmol) and Bent-Gu-CD (40 mg) in 15 mL H_2_O were subjected to ultrasonic irradiation for 15 min. After completion of the reaction, indicated by TLC, Bent-Gu-CD was filtered, washed with MeOH and dried in oven at 70 °C. To examine recyclability of the catalyst, the dried Bent-Gu-CD was applied as a catalyst for eight runs. In Fig. 2, the three ultrasonic-assisted reactions are depicted (Supplementary Fig. S7).

## Conclusion

Covalent composite of Bent and CD is fabricated through reaction of Ben-Cl with IS and GU, followed by reaction with tosylated CD. The catalyst exhibited excellent activity for promoting various chemical reactions, including Knoevenagel condensation, synthesis of xanthan and octahydroquinazolinones in aqueous media under ultrasonic irradiation. It was believed that CD in the framework of the catalytic composite acted as a molecular shuttle for hosting the substrates in its cavity and carrying them to the aqueous media. Bent on the other hand, showed catalytic activity instinctively and rendered the composite heterogeneous. Generality of the protocol, rapidness, facile recovery and recyclability of the catalyst as well as using naturally occurring compounds are other advantageous of the present protocol.

## Supplementary Information

Supplementary Information.
