# Supplementary material for: Composite of bentonite and cyclodextrin as an efficient catalyst for promoting chemical transformations in aqueous media
Source: Sci Rep. 2021 Mar 3;11:5102. doi: 10.1038/s41598-021-84349-9 (PMC7930184; doi:10.1038/s41598-021-84349-9)
Supplement: Supplementary file 1 — Supplementary Information. [file 41598_2021_84349_MOESM1_ESM.docx]

**Supporting information**

**Composite of bentonite and cyclodextrin as an efficient catalyst for promoting chemical transformations in aqueous media**

Fatemeh Koohestani^1^, Samahe Sadjadi^*1^, Majid Heravi^*2^

*^1^Gas Conversion Department, Faculty of Petrochemicals, Iran polymer and Petrochemical Institute, PO Box 14975-112, Tehran, Iran. Tel: +98 2148666 ; Fax: +98 214478, Emai:* [*s.sadjadi@ippi.ac.ir*](mailto:s.sadjadi@ippi.ac.ir)

^2^ *Department of Chemistry, School of Science, Alzahra University, PO Box 1993891176, Vanak, Tehran, Iran. Tel.: +98 21 88044051; fax: +982188041344; Email:* [*m.heravi@alzahra.ac.ir*](mailto:m.heravi@alzahra.ac.ir)

**
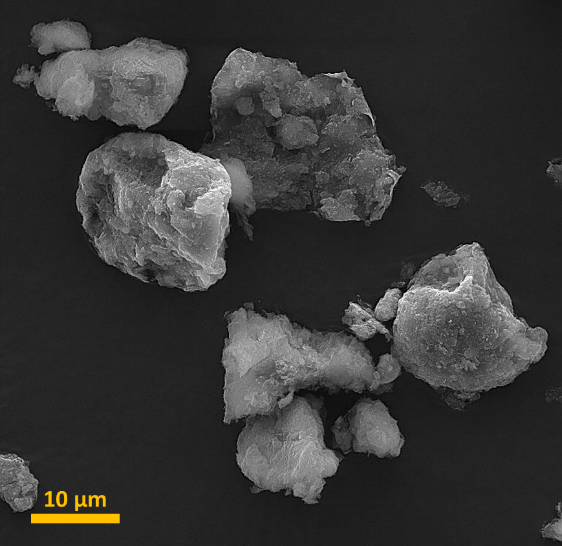
**

**Figure S1.** SEM image of Bent

**Figure S2.** Thermogram of Bent-Gu-CD

**Table S1.** Optimization of amount of catalyst in synthesis of octahydroquinazolinones

| **NO.** | **Catalyst** **amount (mg)** | **Tim**  **(min)** | **Yield %** |
| --- | --- | --- | --- |
| **1** | 10 | 45 | 60 |
| **2** | 20 | 30 | 85 |
| **3** | 30 | 20 | 90 |
| **4** | 40 | 15 | 100 |


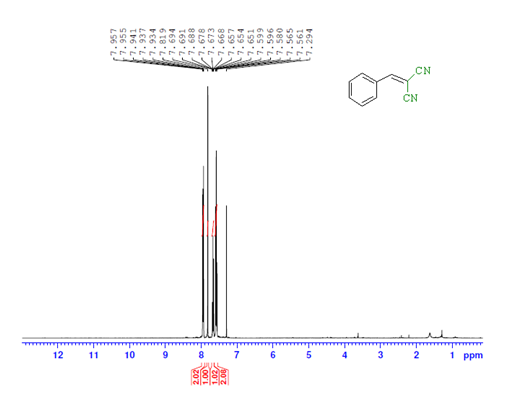


**Figure S3.** ^1^HNMR spectrum of Knoevenagel condensation reaction of malononitrile and benzaldehyde.


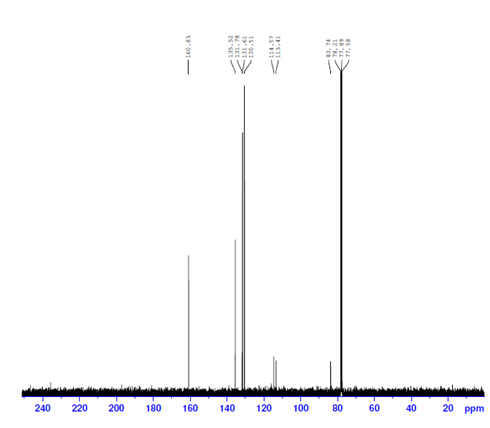


**Figure S4.** ^13^CNMR spectrum of Knoevenagel condensation reaction of malononitrile and benzaldehyde

**Figure S5.** ^1^HNMR spectrum of the model xanthan

**Figure S6.** ^13^CNMR spectrum of the model xanthan


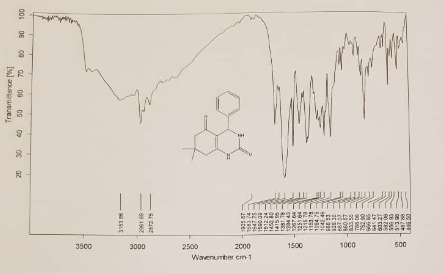


**Figure S7.** FTIR spectrum of the model octahydroquinazolinone
